# The shift in tuberculosis timing among people living with HIV in the course of antiretroviral therapy scale‐up in Malawi

**DOI:** 10.1002/jia2.25240

**Published:** 2019-04-30

**Authors:** Hannock Tweya, Caryl Feldacker, James Mpunga, Henry Kanyerere, Tom Heller, Prakash Ganesh, Dave Nkosi, Mike Kalulu, George Sinkala, Thomas Satumba, Sam Phiri

**Affiliations:** ^1^ The International Union Against Tuberculosis and Lung Disease Paris France; ^2^ Lighthouse Trust Lilongwe Malawi; ^3^ International Training and Education Center for Health University of Washington Seattle WA USA; ^4^ Department of Global Health University of Washington Seattle WA USA; ^5^ National Tuberculosis Control Programme Community Health Science Unit Lilongwe Malawi; ^6^ Bwaila District Hospital Lilongwe Malawi; ^7^ Department of Medicine University of North Carolina School of Medicine Chapel Hill NC USA; ^8^ Department of Public Health College of Medicine School of Public Health and Family Medicine University of Malawi Zomba Malawi

**Keywords:** Antiretroviral therapy, HIV, Tuberculosis, Incidence, Patients living with HIV, Malawi

## Abstract

**Introduction:**

Although the use of antiretroviral therapy (ART) reduces HIV‐associated tuberculosis (TB), patients living with HIV receiving ART remain at a higher risk of developing TB compared to those without HIV. We investigated the incidence of TB and the proportion of HIV‐associated TB cases among patients living with HIV who are receiving ART.

**Methods:**

The study used TB registration and ART programme data collected between 2008 and 2017 from an integrated, public clinic in urban Lilongwe, Malawi. ART initiation was based on either WHO clinical staging or CD4 cell count. The CD4 thresholds for ART initiation eligibility was initially 250 cells/μL then changed to 350 cells/μL in 2011, 500 cells/μL in 2014 and to universal treatment upon diagnosis from 2016. Using TB registration data, we calculated the proportion of TB/HIV patients who were already on ART when they registered for TB treatment by year of TB registration. ART registration data were used to examine TB incidence by calendar year of ART follow‐up and by time on ART.

**Results:**

The overall proportion of TB/patients living with HIV who started TB treatment while on ART increased from 21% in 2008 to 81% in 2017 but numbers remained relatively constant at 500 TB cases annually. The overall incidence rate of TB among patients on ART was 1.35/100 person‐years (95% CI 1.28 to 1.42). The incidence of TB by time on ART decreased from 6.4/100 person‐years in the first three months of ART to 0.4/100 person‐years after eight years on ART. TB incidence was highest in the first month on ART. The annual rate of TB among patients on ART rapidly decreased each calendar year and stabilized at 1% after 2013. Although the risk of developing TB decreased with year of ART initiation in univariable analysis, there was no significant association after adjusting for sex, age and reason for ART eligibility.

**Conclusions:**

The decline in TB incidence over calendar years suggests protective effects of early ART initiation. The high TB incidence within the first month of ART highlights the need for more sensitive tools such as X‐ray and GeneXpert to identify patients living with HIV who have clinical and subclinical TB disease at ART initiation.

## 1 Introduction

Despite recent declining incidence, tuberculosis (TB) is the leading cause of morbidity and mortality among people living with HIV 1. In 2015, an estimated 1.2 million TB cases occurred among people living with HIV; TB accounted for a third of AIDS‐related deaths 1. The World Health Organization (WHO), therefore, recommends strengthened TB/HIV service delivery based on key improvements in TB case finding and treatment, TB infection control, isoniazid preventive therapy (IPT) and early antiretroviral therapy (ART) initiation 2, 3.

The use of ART reduces the risk of HIV‐associated TB by up to 67% 4. However, this reduction is time dependent: TB incidence is highest in the first three months of ART and decreases gradually during the first two to three years on ART 5, 6, 7. The high TB incidence in early months on ART is at least partly due to unmasking of subclinical TB because of initial restoration of immune response 5, 8. Early initiation of ART reduces mortality, morbidity and HIV‐associated TB 9, 10. However, long‐term TB incidence among people on ART remains high: those on ART are several times more likely to get TB than non‐HIV‐infected populations in the same communities 11, 12. Increasing numbers of HIV‐infected people on ART may create populations with long‐term heightened susceptibility to TB, potentially contributing significantly to the overall TB burden 11. In Malawi, the proportion of co‐infected TB/HIV patients registering for TB treatment while on ART increased from 52% (TB cases: 1526) in 2011 to 92% (TB cases: 1819) in 2017 13, 14. The reasons for this dramatic shift in TB cases among those already on ART remain unclear. Therefore, we explored (Q1) the overall trends of HIV‐associated TB cases attributed to patients living with HIV who are on ART by year of TB treatment registration; (Q2) TB incidence trends among those on ART by calendar year of ART follow‐up; and (Q3) TB incidence by patient's time on ART. Better understanding of these trends may lead to development of interventions that further reduce HIV‐associated TB among individuals on ART.

## 2 Methods

### 2.1 Study design and Setting

This cross‐sectional and retrospective cohort study used routine TB and ART programme data from an integrated, public clinic, Martin Preuss Centre (MPC) in urban Lilongwe, Malawi. MPC has three units: HIV testing and counselling, TB and ART. MPC clinic services were detailed previously 15. In brief, MPC registers 1200 TB cases annually. Since 2008, 97% of TB cases know their HIV status before starting TB treatment. Individuals were screened for TB‐associated symptoms. The initial diagnostic test for presumptive TB case was sputum smear microscopy or chest radiography depending on the clinical presentation. Diagnosis of extrapulmonary TB was mostly based on radiography but it was also diagnosed bacteriogically or histopathologically. In 2015, rapid molecular test (Xpert MTB/RIF) and MGIT liquid culture were introduced for screening retreatment cases, hospitalized TB suspects, multidrug‐resistant TB suspects and patients living with HIV. Since 2016, screening for disseminated TB in patients with advanced immunosuppression was routinely done using urine lipoarabinomannan (LAM) 16 and FASH 17. TB diagnosis information, HIV and ART status at TB treatment registration were recorded in a national TB register. TB/HIV patients already on ART at TB treatment registration either continued with ART treatment at the previous facility or transferred to receive ART at MPC. TB/HIV‐positive patients who received ART at MPC were registered in an electronic medical record system (EMRs) and expected to initiate ART (within two months between 2008 and 2011, and within two weeks thereafter).

All HIV‐infected individuals without a diagnosis of active TB were registered in the EMRs. Previously, eligibility for ART initiation was based on either WHO clinical staging (3 and 4) or CD4 cell count if a patient had WHO stage 1 or 2 condition 13, 18, 19. CD4 cell count cutoff used for ART eligibility was raised from ≤250 to ≤350 cells/μL in 2011 and ≤500 cells/μL in 2014. Since 2011, HIV‐infected pregnant and lactating women were initiated on ART regardless of WHO clinical stage and immunological status 13. From July 2016 onwards, all HIV‐infected individuals initiated ART according to test‐and‐treat policy 20. ART follow‐up visits were scheduled monthly for the first six months and every two or three months thereafter. TB screening was done at each ART follow‐up visit using a symptom checklist; those with TB symptoms were referred for further TB investigations. Only pre‐ART patients received IPT until June 2017. From July 2017, IPT was offered to all ART patients for life at the study facility. ART treatment outcomes (alive, stopped ART and transfer‐out) were updated in the EMRs at each clinic visit; treatment outcomes such as lost to follow‐up (LTFU) and died were updated retrospectively.

### 2.2 Variable definitions

At TB registration, HIV status was classified as “HIV‐infected,” “HIV‐negative” and “Unknown HIV status.” ART status was classified as either “Already on ART” (previously registered for ART) or “Not on ART” (not already registered for ART). At ART registration, patients were classified as having TB if the patient had either an existing clinical episode of TB at the time of ART initiation/any clinical episode of TB within 14 days after ART initiation.

### 2.3 Study population and data sources

Two population groups were analysed. First, to explore overall trends of HIV‐associated TB cases attributed to HIV‐infected patients on ART by year of TB treatment registration, we abstracted data from TB registers for adult HIV‐negative and HIV‐infected TB patients (aged ≥15 years) who registered for TB treatment between 1 January 2008 and 31 December 2017 regardless of where they accessed ART. Using a sub‐population of TB registrations who initiated ART at MPC, we explored trends of HIV‐associated TB cases among patients living with HIV according to time on ART by year of TB treatment registration. Second, to assess TB incidence by time on ART and calendar year, data were extracted from the ART EMRs, including all adult HIV‐infected individuals on ART at MPC regardless of TB status at ART initiation. ART patients were excluded from analysis of TB incidence if they had only one ARV dispensing visit or dropped‐out (transferred ART care, died LTFU) before completing TB treatment.

### 2.4 Statistical analysis

To investigate trends of TB cases attributed to patients living with HIV who are on ART (Q1), we calculated the proportion of TB/HIV patients already on ART at TB registrations by year of TB treatment registration. Second, we examined TB incidence by calendar year of ART follow‐up and by time on ART among all adult HIV‐infected individuals on ART at MPC regardless of TB status at ART initiation (Q2 and Q3). For patients with TB at ART registration, person‐time at risk of developing active TB began on the date they completed TB treatment while those without TB, person‐time at risk of TB began on the date of ART initiation. The time at risk of TB ended at death, transfer‐out, LTFU or censored on 31 December 2017. Multiple TB episodes per patient were allowed. Person‐times accrued during treatment of a TB episode while on ART were excluded from calculation of TB incidence rates. To assess the effect of year of ART initiation on TB incidence, Cox models estimated unadjusted and adjusted hazard ratios (HR) of risk for active TB by period of ART initiation. Models were adjusted for sex, age at ART initiation and reason for ART eligibility.

### 2.5 Ethical approval

This retrospective study of already collected anonymous data received both approval and consent waiver from the Malawi National Health Science Research Committee in Lilongwe, Malawi.

## 3 Results and discussion

Of 22,570 TB cases notified, 13,808 were HIV positive at TB treatment registration. The prevalence of HIV among TB cases declined slightly from 63% in 2008 to 58% in 2017.

**Figure 1:**
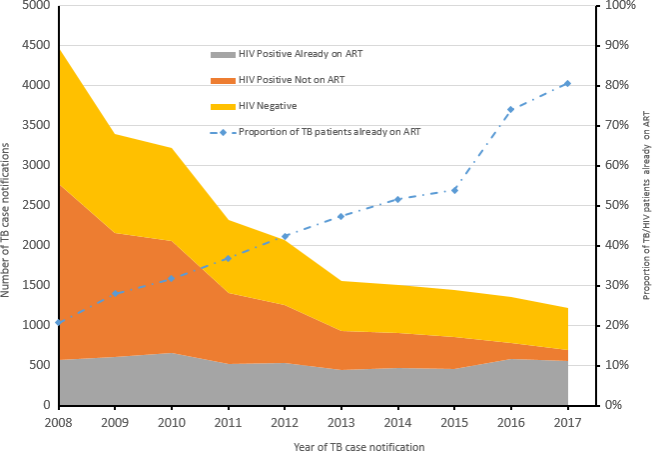
Overall trends of TB case notifications and antiretorviral therapy status among TB patients registering at Martin Preuss Center in Lilongwe, Malawi. TB, tuberculosis.

Overall absolute number of TB/HIV co‐infected cases notified decreased from 1455 in 2008 to 511 in 2017 (Figure 1). Overall proportion of HIV‐infected TB patients already on ART at TB treatment registration increased from 21% to 81% while absolute numbers remained relatively constant at 500 TB cases annually.

Of the 13,808 TB/HIV patients, 5719 (41%) registered for ART at MPC: 3000 (52%) before initiating ART and 2719 (48%) after initiating ART. Of the 2719 TB cases diagnosed after ART, most cases occurred between zero and three months of ART initiation (Table 1). From 2008 to 2014, the number of ART patients registering for TB treatment increased, followed by steep decline between 2015 and 2017. The proportion of ART patients registered for TB treatment between four and eleven months on ART remained relatively constant across years. However, the proportion of ART patients registered for TB treatment after one or more years on ART increased gradually from 2% in 2009 to 39% in 2017.

**Table 1:** Trends of TB case notifications for patients who received ART services at Martin Preuss Center in Lilongwe, Malawi

TB registration Year	Total	Started TB treatment before ART initiation (n=3000)	Started TB treatment after ART initiation (n=2719)		
		Zero to three months, n (%)		Four to eleven months, n (%)	Twelve months +, n (%)
Total	5719	3000 (52)	1780 (31)	302 (5)	637 (11)
2008	708	632 (89)	56 (8)	20 (3)	0 (0)
2009	673	527 (78)	101 (15)	30 (4)	15 (2)
2010	827	564 (68)	184 (22)	34 (4)	45 (5)
2011	739	474 (64)	155 (21)	49 (7)	61 (8)
2012	656	301 (46)	234 (36)	45 (7)	76 (12)
2013	446	168 (38)	202 (45)	17 (4)	59 (13)
2014	441	125 (28)	245 (56)	16 (4)	55 (12)
2015	500	127 (25)	255 (51)	28 (6)	90 (18)
2016	423	61 (14)	214 (51)	32 (8)	116 (27)
2017	306	21 (7)	134 (44)	31 (10)	120 (39)

ART, antiretroviral therapy; TB, tuberculosis.

**Figure 2:**
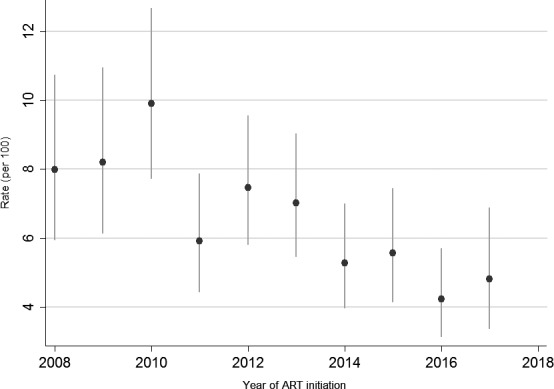
Rates of TB cases per 100 person‐years with 95% confidence interval within the first three months on ART. ART, antiretroviral therapy; TB, tuberculosis.

At MPC, 35,983 patients living with HIV registered for ART: 881 (2%) were excluded from analysis of incidence TB due to drop‐out. Among the other 35,102 ART patients, the median follow‐up from ART initiation was 2.0 years (interquartile range 0.7 to 4.7); 1428 (4%) developed active TB. The annual rate of TB among those on ART decreased rapidly from 6.0% in 2008 (95% CI 4.7 to 7.6 and 1.1% in 2013 (95% CI 0.9 to 1.2), a reduction of 82%. Thereafter, the annual rate of TB remained constant at 1 per 100 person‐years. Looking at TB rates within the first three months of ART by calendar year, rates peaked in 2010, thereafter decreasing by 57% from 8.9% (95% CI 7.1 to 11.2) to 3.8% (95% CI 2.7 to 5.4) in 2017 (Figure 2). In univariable analysis, with those initiating ART in 2008 to 2010 as reference, TB hazard declined over time: 2011 to 2013: HR 0.76 (95% 0.67 to 0.86); 2014 to 2015: HR 0.72 (0.62 to 0.84); 2016 to 2017: HR 0.60 (95% 0.50 to 0.73). After adjusting for sex, age at ART initiation and reason for ART eligibility, year of ART initiation was not associated with risk of TB.

The overall TB incidence on ART was 1.35 cases per 100 person‐years (95% CI 1.28 to 1.42). The highest TB incidence was observed in the first three months of ART (6.4/100; 95% CI 5.9 to 7.0). TB incidence dropped nearly sixfold between month one and two years on ART (1.1/100; 95% CI 0.9 to 1.2). Thereafter, TB risk declined slowly over time on ART, to 0.6 (95% CI 0.4 to 0.7) at four years on ART. Among patients on ART for one year or more, the annual rate of TB declined from 1.4% (95% CI 0.8 to 2.3) in 2009 to 0.4% in 2014 (95% CI 0.3 to 0.6) then increased to 0.7% in 2016 (95% CI 0.6 to 0.9; Table 2).

**Table 2:** Rate of TB per 100 person‐years among HIV‐infected patients who were on ART for one year or more at Martin Preuss Centre clinic in Lilongwe, Malawi

Calendar year	TB cases	Person‐years	Rate (95% confidence interval)
2008	‐	‐	‐
2009	13	96	1.35 (0.79 to 2.33)
2010	40	295	1.35 (0.99 to 1.85)
2011	52	497	1.05 (0.80 to 1.37)
2012	66	694	0.95 (0.75 to 1.21)
2013	51	906	0.56 (0.43 to 0.74)
2014	46	1074	0.43 (0.32 to 0.57)
2015	81	1242	0.65 (0.52 to 0.81)
2016	104	1378	0.75 (0.62 to 0.91)
2017	96	1270	0.76 (0.62 to 0.92)

ART, antiretroviral therapy; TB, tuberculosis.

The study showed that rates of TB within the first three months of ART and annual TB rates decreased significantly by 57% and 82%, respectively, during the study period. Annual TB rates among those on ART stabilized at 1% after 2013. However, the proportion of ART patients who started TB treatment increased fourfold during the study period. There were significant increases among those who started TB treatment within three months on ART and after one year on ART. Although these findings highlight major gains in TB control, TB remains a major challenge for HIV‐infected people, even in the era of increasing ART access. We highlight two primary observations in TB control and discuss possible interventions.

First, TB incidence declined dramatically within the first three months on ART and over calendar years possibly due to early initiation of ART 10. As the Malawi HIV programme raised the CD4 thresholds for ART eligibility from 250 to 350 cells/μL in 2011 to 500 cells/μl in 2014 13, 18, 19, a clear trend in declining annual TB rates among ART patients was observed between 2008 and 2014. Overall TB rates in the first three months of ART also declined with the implementation of test‐and‐treat policy. Initiating more patients living with HIV with less advanced HIV disease, as recommended by WHO 21 and confirmed in this study, should significantly reduce TB incidence among patients living with HIV. However, in spite of the declines, incidence remained high possibly due to unmasking of subclinical TB 5, 8 and misdiagnosing at ART initiation. Systematic routine use of symptomatic TB screening criteria and highly sensitive TB screening tools as GeneXpert, X‐ray and LAM assays are needed to identify patients living with HIV who have clinical and subclinical TB disease.

Second, the proportion of all TB cases attributed to ART patients increased by fourfold while the absolute numbers of TB cases remained relatively constant over the study period. Similar trends were reported in Malawi TB data: the proportion of HIV‐infected TB patients on ART at TB treatment registration increased from 52% in 2011 to 92% in 2017 13, 14. Increases in HIV testing combined with early linkages to ART and more patients developing TB one year on ART could explain the trends. Declining TB incidence within the three months of ART initiation resulted from HIV‐infected patients starting ART before developing TB. The annual TB incidence among patients on ART more than one year increased from 0.4% to 0.7% between 2014 and 2017. The rate of TB observed among ART patients after four years on ART (500 per 100,000 persons) was three times higher than in the general Malawi population, estimated at 159 per 100,000 population 22. The high TB incidence and increasing trend in the number of ART patients developing TB after one year on ART also provides empirical evidence that ART, alone, is not enough to prevent TB among HIV‐infected patients.

These findings have important programmatic implications. First, as HIV‐infected individuals remain at a higher risk of developing TB than people without HIV, and patients living with HIV with high viral loads are at increased risk of developing TB 23, HIV programmes should strengthen comprehensive HIV efforts including increased ART adherence to prevent ongoing viral replication, regular viral load monitoring and timely introduction of second‐line treatment among those failing ART. Second, other TB control interventions such as intensified TB case finding and IPT are necessary to reduce the risk of TB further among ART patients. There is enough evidence that prolonged use of IPT along with ART has an additive effect on reducing TB incidence 24, 25, 26, 27. Considering long‐term heightened susceptibility to TB in the study population, Malawi prioritized the provision of life‐long IPT to patients living with HIV in selected districts.

Our study has several limitations. First, CD4 counts and viral load were not available for most patients, reducing exploration of variable's effects on TB. Second, although TB diagnosis was done according to national TB guidelines, some cases might have been missed, potentially underestimating the incidence of TB. Third, due to limited data, we could not assess effects of intensified TB case finding and TB diagnostic services (e.g. introduction of GeneXpert in 2015) on TB incidence. Despite these limitations, we believe the strengths of these results outweigh the known data weaknesses.

## 4 Conclusions

Overall, TB incidence declined among those on ART by calendar year of ART follow‐up suggests the successes of both Malawi's TB control and early ART initiation effort. Continued focus on early ART initiation will reduce TB incidence further. However, the long‐term risk of incident TB among patients on ART and increasing proportion of TB cases attributed by HIV‐infected patients receiving ART are concerning. The high TB incidence within the first month of ART highlights the need for sensitive diagnostic screening for subclinical TB such as X‐ray and GeneXpert at ART initiation. Intensified TB case finding, improved ART adherence, timely ART drug substitution and provision of IPT could merit inclusion in comprehensive TB/HIV programs.

## Competing interests

The authors declare that they have no competing interests.

## Authors’ contributions

HT conceived the study and designed the study protocol; HT, MK, HK, JM, DN, GS, TS, TH and SP implemented the study. HT and CF carried out analysis and interpretation of these data, and. drafted the manuscript; MK, HK, JM, DN, GS, TH, TS and SP critically revised the manuscript for intellectual content. All authors read and approved the final manuscript. HT and SP are guarantors of the paper.
